# Curcumin Enhanced Ionizing Radiation-Induced Immunogenic Cell Death in Glioma Cells through Endoplasmic Reticulum Stress Signaling Pathways

**DOI:** 10.1155/2022/5424411

**Published:** 2022-10-04

**Authors:** Zenghe Xiu, Ting Sun, Ying Yang, Yuping He, Shuangyu Yang, Xuefei Xue, Wei Yang

**Affiliations:** ^1^State Key Laboratory of Radiation Medicine and Protection, School of Radiation Medicine and Protection, Collaborative Innovation Center of Radiation Medicine of Jiangsu Higher Education Institutions, Soochow University, Suzhou, Jiangsu, China; ^2^Neurosurgery and Brain and Nerve Research Laboratory, The First Affiliated Hospital of Soochow University, Suzhou, Jiangsu, China

## Abstract

**Objective:**

Local radiotherapy may cause distant tumor regression via inducing immunogenic cell death (ICD). Here, we investigated the effect of curcumin on ionizing radiation-induced immunogenic cell death in normoxic or hypoxic glioma cells and its mechanism in vitro and vivo.

**Methods:**

Hypoxic or normoxic glioma cell apoptosis and the cell surface exposure of calreticulin (CRT) were detected by flow cytometry. Extracellular ATP and HSP70 were measured by chemiluminescence assay and ELISA, respectively. Endoplasmic reticulum (ER) stress protein levels were detected by western blot. Moreover, the induction of bona fide ICD was detected by vaccination assays in mice bearing glioma model. Spleen lymphocytes and tumor-infiltrating lymphocyte subsets were analyzed by flow cytometry and immunohistochemistry.

**Results:**

Curcumin incubation before X-ray irradiation significantly increased radiation-induced apoptosis rate in normoxic or hypoxic glioma cells. Curcumin enhanced radiation-induced CRT exposure, release of HSP70 and ATP, and ER stress signaling activity. After treatment with ER stress pathway inhibitors, cell apoptosis and CRT exposure induced by the combination treatment of curcumin and X-ray were reduced. In vaccination experiments, glioma cells irradiated by X-ray produced a strong immunogenic response rejecting tumor formation in 70% mice. In comparison, cells treated by curcumin and X-ray produced a stronger immune response rejecting tumor formation in 90% mice. The combination treatment increased the percentage of tumor-infiltrating CD4+, CD8+ T lymphocytes, and CD11c+ dendritic cells compared to X-ray irradiation alone.

**Conclusion:**

Ionizing radiation-induced normoxic or hypoxic glioma immunogenic cell death could be further enhanced by curcumin through activating the ER stress PERK-eIF2*α* and IRE1*α*-XBP1 signaling pathways.

## 1. Introduction

Glioma is the deadliest subtype of primary brain tumor, whose overall survival is about 15 months [1, 2]. Even though glioma is treated with the combination of conventional therapies, the efficacy is still limited. Hypoxia exists in almost all solid tumors, including glioma. DNA damage resistance and oxidative stress resistance caused by hypoxia can significantly reduce cancer cell death induced by radiotherapy [3, 4].

Immunogenic cell death (ICD) is different from general apoptosis. Some physical factors, including ionizing radiation (IR), and chemical factors induce endoplasmic reticulum (ER) stress via reactive oxygen species (ROS), leading to the cell surface exposure or release of damage-associated molecular patterns (DAMPs) and triggering antitumor immune response [5, 6]. DAMPs are endogenous molecules; however, when they are exposed to the surface of dying cells or released, they can perform immunoregulatory functions. There are some DAMPs closely related to ICD. ER chaperone calreticulin (CRT) exposed on cell surface, an “eat me” signal, can be recognized by phagocytic cell surface CD91 receptor, thereby promoting the phagocytosis of dying cells [7]. Other cytoplasmic or ER chaperone molecules, such as heat shock protein (HSP) 70/90, can also act as an “eat me” signal through cell membrane exposure [8]. ATP acts as a strong “find-me” signal via binding to P2Y2 purine receptor to attract monocytes or acts as a proinflammatory molecule to bind to P2X7 receptor on innate immune cells [9]. On the contrary, CD47, a “don't eat me” signal, is a transmembrane protein that can bind to a variety of ligands and regulate tumor development [10].

Curcumin, an active phenolic substance, is a multifunctional molecule with antioxidant, antibacterial, anti-inflammatory, and immunomodulatory effects. Curcumin can regulate cell apoptosis via various pathways and affect the metastasis and proliferation of tumors [11]. Curcumin can suppress HIF-1 activity in a variety of tumor cells and improve tumor hypoxia [12, 13].

In this study, to investigate the effect of curcumin on the IR-induced immunogenic cell death in glioma cells and its mechanism, we detected the apoptosis of human glioma U251 cells and mouse glioma GL261 cells, the release of ICD-related DAMPs, and the activation of ER stress pathways after the combination treatment of curcumin and X-ray irradiation under normoxic and hypoxic condition. Furthermore, animal models using C57BL/6J mice implanted with GL261 cells were conducted to verify whether curcumin could enhance IR-induced ICD in vivo through ER stress pathways.

## 2. Materials and Methods

### 2.1. Cell Culture

Mouse glioma GL261 and human glioma U251 cell lines were purchased from the Shanghai Institutes for Biological Sciences, Chinese Academy of Sciences, and were cultured in DMEM with 10% FBS. Hypoxia cells were treated with 1% O_2_, 5% CO_2_, and 94% N_2_ in a hypoxia workstation (Invivo2 1000, Ruskinn).

### 2.2. X-Ray Irradiation

Cells were exposed to 10 Gy (normoxia)/25 Gy (hypoxia) X-ray (160 kV, 1.16 Gy/min) by a linear accelerator (RadSource, RS2000) at room temperature.

### 2.3. Flow Cytometry

Cells were digested and collected after curcumin and IR treatment. The spleen and tumor were cut into tissue pieces, and cell suspension was made with trypsin. Cells were stained using PE-Annexin V/7-AAD apoptotic Kit (BioLegend) according to the manufacturer's instructions. Cells were incubated with antibodies for 30 min at room temperature. The primary antibodies include PE-conjugated anti-CRT rabbit antibody (1 : 50, Cell Signaling), anti-CD47 antibody (1 : 100, Biorbyt), FITC-conjugated anti-CD45 mouse antibody (1 : 100, eBioscience), APC-conjugated anti-CD3 mouse antibody (1 : 100, eBioscience), and V450-conjugated anti-CD8 mouse antibody (1 : 100, eBioscience). Labeled cells were analyzed by a flow cytometric Becton Dickinson FACScan (BD Biosciences).

### 2.4. Calreticulin Exposure

Cells were stained with Calreticulin-PhycoErythrin antibody (1 : 50, Cell Signaling) and then washed and resuspended in FACS buffer for assessing with flow cytometry. For confocal microscopy (Olympus FV1200), 3 × 10^5^/ml cells were seeded on sterile coverslips and incubated with CRT antibody (1 : 1000, Cell Signaling) for 12 h, then incubated with Alexa Fluor 555 secondary antibodies and Hoechst 33342 (Beyotime) for 1 h, and assessed by confocal microscopy. The wavelengths of excite light and emit light are 488 nm and 575 nm, respectively.

### 2.5. Western Blot

Cell lysates were prepared in RIPA lysis buffer containing phenylmethylsulfonyl fluoride (PMSF). The protein samples were separated by 10% sodium dodecyl sulfate-polyacrylamide gel (SDS-PAGE) and then were transferred onto the polyvinylidene fluoride (PVDF) membrane. The membrane was then blocked with 5% nonfat dry milk for 1 h. Primary rabbit antibodies include anti-*β*-actin (1 : 1000, Cell Signaling), anti-CHOP (1 : 1000, Cell Signaling), anti-PERK (1 : 1000, Cell Signaling), phospho-PERK (1 : 1000, Invitrogen), IRE1-*α* (1 : 1000, Cell Signaling), p-IRE1-*α* (1 : 1000; Invitrogen), and splicing XBP1 (1 : 1000, Cell Signaling). HRP-conjugated anti-rabbit secondary antibodies were used, and the chemiluminescent signal was detected by using electrochemiluminescence (ECL) reagents.

### 2.6. Enzyme-Linked Immunosorbent Assay (ELISA)

After irradiation, glioma cells were cultured in normoxia or hypoxia for 24 h, and then, the cell culture supernatants were centrifuged at 500 g for 5 min at room temperature and stored at -80°C. The extracellular HSP70 protein contents were detected using a HSP70 ELISA kit (Shanghai sig Biotechnology Co., Ltd.) according to the corresponding manufacturer's instructions.

### 2.7. ATP Assays

Supernatants of irradiated glioma cells were used to assess extracellular ATP with a luciferase assay kit (Beyotime, Shanghai) following the manufacturer's instructions. In brief, ATP detection working solutions were prepared and seeded (100 *μ*l/well) into 96-well plates. Then, 50 *μ*l of standard or sample was added into each well. The plates were put into the microplate reader, and the detector is 1 mm away from the liquid level to detect the luminescence value.

### 2.8. DC Generation and Maturation

Peripheral blood was collected from healthy donors and PBMCs were isolated by gradient centrifugation at 400-500 g for 20-25 min at 18-20°C. PBMCs were resuspended and seeded (2 × 10^6^ cells/ml/well) into 6-well plates. After incubation in 37°C for 2 h, nonadherent cells were removed, and complete medium (10% FBS, 800 U/ml GM-CSF, 500 U/ml IL-4 growth factor) was provided. The medium was replaced every 2-3 days. On the 6th day, 10 ng/ml TNF-*ɑ* was added to the medium, and then, mature DCs were harvested 2 days later.

### 2.9. DC Chemotaxis Assay

U251 cells were seeded (1 × 10^5^ cells/500 *μ*l) in the lower chamber after 10 Gy or 25 Gy X-ray irradiation. Mature DCs (3 × 10^5^ cells/200 *μ*l) were added into the upper chamber (8.0 *μ*m, LABSELECT, China). After incubation for 12 h and 24 h, DCs attached to the bottom of the upper chamber were fixed with 4% paraformaldehyde and stained with 0.1% crystal violet.

### 2.10. DC Phagocytosis Assay

U251 cells incubated with or without 15 *μ*M curcumin were marked red by CellTrace kit (Invitrogen) according to the instruction manual. Mature DCs were stained green using the same kit. U251 cells (2 × 10^4^ cells/500 *μ*l) and mature DCs (4 × 10^4^ cells/500 *μ*l) were mixed together and incubated in 35 mm dishes for 8 h.

### 2.11. Prophylactic Vaccinations

The 6-8-week-old male C57BL/6J mice with the body weight of 18-20 g (Experimental Animals Center of Shanghai Institute of Life Science) were fed and housed in SPF condition. The mice were randomly divided into control group, curcumin group, X-ray treatment group, and curcumin combined with X-ray treatment group. GL261 cells were treated with PBS, curcumin (30 *μ*M), X-ray (10 Gy), and X-ray (10 Gy) and curcumin for 24 h, respectively. Then, 1 × 10^6^ GL261 cells were injected into the left hind limb of the mice subcutaneously. One week later, 3 × 10^6^ live GL261 cells were injected into the right hind limb subcutaneously. The tumor volumes at the right were measured with calipers every three days and calculated as (length^2^ × width)/2, and the tumor formation rates were also calculated.

### 2.12. Hypoxic Transplanted Tumor Model

For in vivo implantation, GL261 cells were injected subcutaneously at 3 × 10^6^ cells in 0.1 ml PBS in the right hind limb of male C57BL/6J mice. Cobalt chloride was used to simulate hypoxia in xenograft tumors in vivo. After tumorigenization of mice, CoCl_2_ aqueous solution with a concentration of 260 mg/l was prepared as drinking water for mice. When the diameter of tumor reached about 6-8 mm, the hypoxic transplanted tumors were subjected to 25 Gy X-ray irradiation (6 MV, 100 cGy/min) by a PRIMUS accelerator (SIEMENS Medical Solutions, Erlangen, Germany) at room temperature, while the normoxic transplanted tumors were subjected to 10 Gy X-ray irradiation. The mice in combined therapy group were intraperitoneally injected with 200 *μ*l curcumin (50 *μ*M) 24 hours before irradiation. All animal experimental protocols were approved by the institutional animal care and use committee and complied with the code of ethics for animal experimentation.

### 2.13. Immunohistochemical (IHC) Staining

The mice were sacrificed 48 hours after irradiation. Tumor tissues and spleen tissues were fixed in paraffin, imbedded, and cut for 4 mm sections. Tumor sections were incubated with primary antibodies, including anti-mouse CD4 (1 : 100, Cell Signaling), CD8 (1 : 100, Cell Signaling), CD11c (1 : 100, Cell Signaling), FoxP3 (1 : 100, Cell Signaling), CHOP (1 : 100, Cell Signaling), phospho-PERK (1 : 100, Invitrogen), p-IRE1-*α* (1 : 100; Invitrogen), and splicing XBP1 (1 : 100, Cell Signaling) antibodies, at 4°C overnight, and biotin-labeled secondary antibody for 30 min at 37°C. The final signal was developed using the 3,3′-diaminobenzidine (DAB) substrate, and the sections were observed under optical microscope. The percentage of positive cells was calculated as number of positive (brown) cells/total number of cells × 100 in 9 randomly selected fields (400×).

### 2.14. Statistical Analysis

GraphPad Prism 8 software was used for statistical analyses. The unpaired two-tailed *t*-test was used for comparison of data between two groups. For comparisons among more than two groups, one-way analysis of variance (ANOVA) followed by the Bonferroni posttest was performed. *P* < 0.05 was considered statistically significant.

## 3. Results

### 3.1. Curcumin Enhanced Apoptosis Induced by IR in Normoxic and Hypoxic Glioma Cells

**Figure 1:**
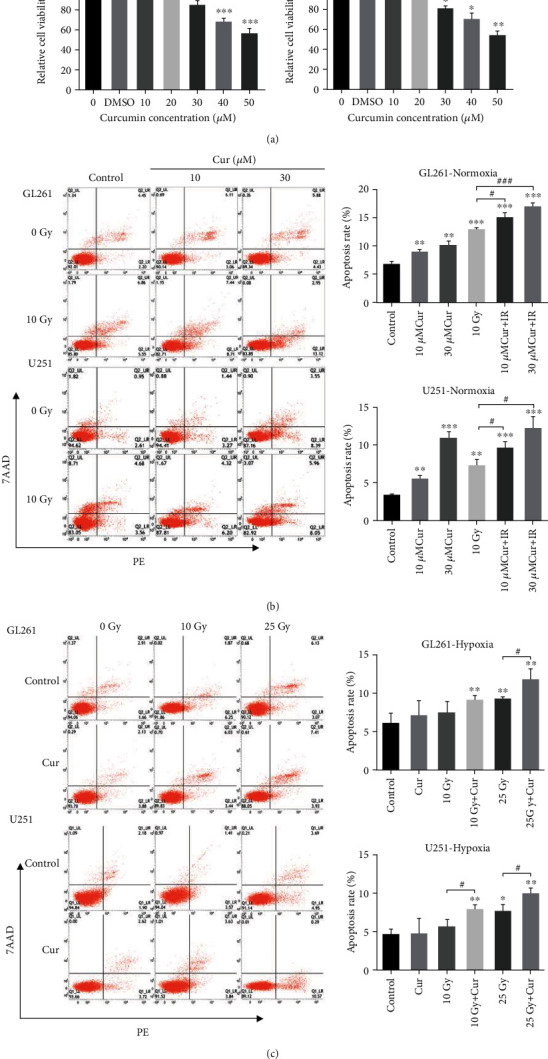
Curcumin enhanced apoptosis induced by IR in normoxic and hypoxic glioma cells. (a) The survival rates of GL261 and U251 cells after treatment with various concentrations of curcumin for 24 h. (b) Under normoxic conditions, GL261 and U251 cells were treated with 10 and 30 *μ*M curcumin and incubated for 24 h after 10 Gy IR. Cell apoptosis was detected by flow cytometry after 7AAD/PE staining. (c) Under hypoxic conditions, GL261 and U251 cells were treated with/without 30 *μ*M curcumin and incubated for 24 h after 10 Gy/25Gy IR. Cell apoptosis was assessed by flow cytometry after 7AAD/PE staining. ^∗^*P* < 0.05, ^∗∗^*P* < 0.01, and ^∗∗∗^*P* < 0.001 vs. control; ^#^*P* < 0.05 and ^###^*P* < 0.001 vs. IR.

GL261 and U251 cells were treated with various concentrations of curcumin for 24 h. The viability of glioma cells decreased (Figure 1(a)), while the apoptosis rate of glioma cells increased (Figures 1(b) and 1(c)) with the increase of curcumin concentration. 10 Gy IR alone could induce apoptosis of normoxic glioma cells but could not significantly induce apoptosis of hypoxic glioma cells. Compared with 10 Gy IR alone, treatment with 30 *μ*M curcumin for 24 h before irradiation significantly increased the apoptosis of normoxic or hypoxic glioma cells. For hypoxic glioma cells, the apoptosis rate induced by 10 Gy IR+30 *μ*M curcumin was similar to that induced by 25 Gy IR alone.

### 3.2. Curcumin Enhanced IR-Induced ICD-Related DAMP Exposure or Release

**Figure 2:**
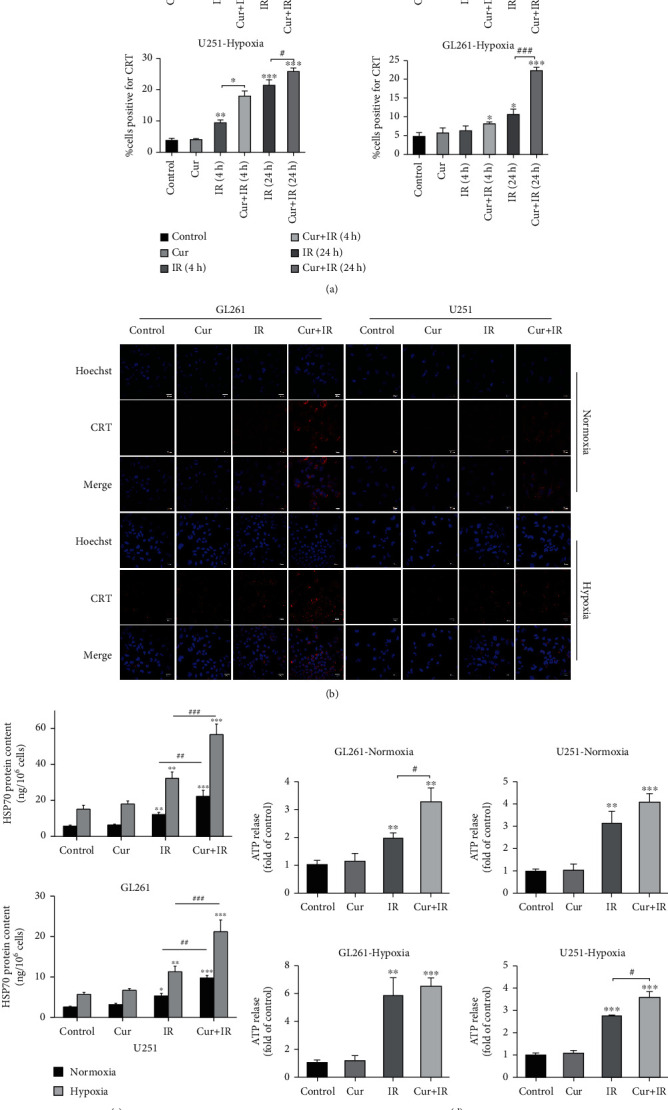
Curcumin enhanced IR-induced ICD-related DAMPs exposure or release. CRT exposure was assessed by flow cytometry (a) and immunofluorescence (b). HSP70 release (c) and ATP release (d) were detected by ELISA and chemiluminescent assay, respectively. (e) CD47 exposure was assessed by flow cytometry. ^∗^*P* < 0.05, ^∗∗^*P* < 0.01, and ^∗∗∗^*P* < 0.001 vs. control; ^#^*P* < 0.05, ^##^*P* < 0.01, and ^###^*P* < 0.001 vs. IR.

We detected CRT exposure on glioma cell surface using flow cytometry and immunofluorescence assay, respectively. The data showed CRT exposure on the surface of the cell membrane can be induced by IR. It occurred as early as 4 h after IR and was more significant at 24 h after IR (Figure 2(a)). Curcumin alone did not induce CRT cell surface exposure, whereas in normoxic and hypoxic glioma cells treated with curcumin prior to IR, CRT exposure was enhanced compared to IR alone (Figure 2(b)). Further, we assessed the release of ATP and HSP70 at 24 h after IR by chemiluminescent assay and ELISA, respectively. Compared with IR, both the extracellular ATP and HSP70 were increased in the curcumin+IR group under normoxic or hypoxic condition (Figures 2(c) and 2(d)). These data indicated that the combination treatment of curcumin and IR promoted ATP and HSP70 release from the dying cells. In addition, CD47 on cell membrane was declined after combination treatment in normoxic and hypoxic glioma compared to IR alone (Figure 2(e)).

### 3.3. Curcumin Enhanced IR-Induced ICD by Activating ER Stress

**Figure 3:**
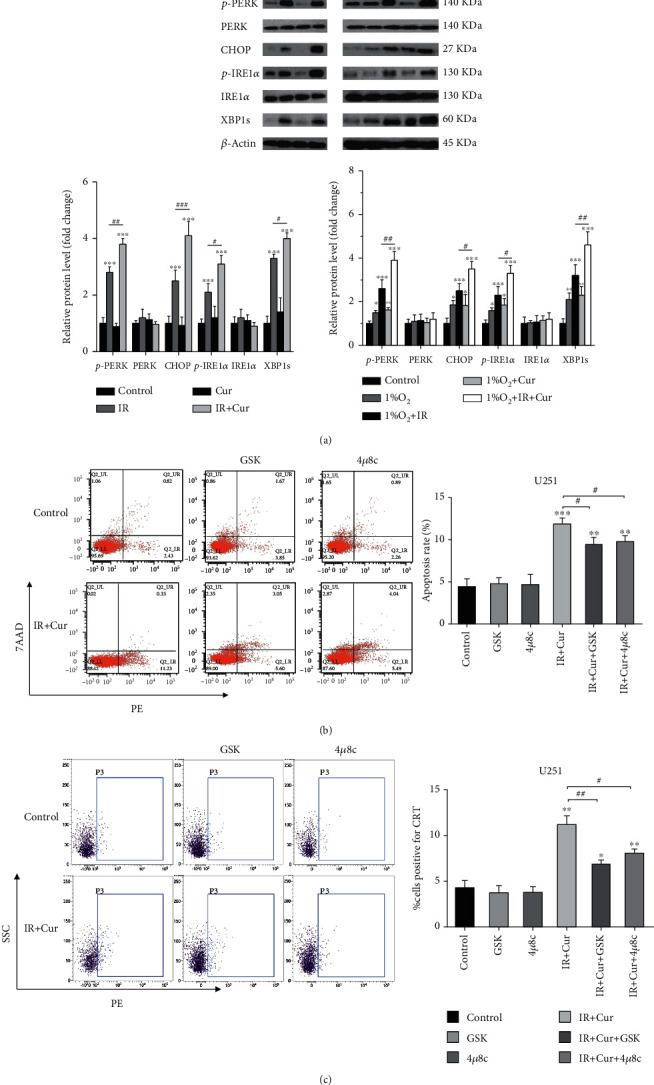
Curcumin enhanced IR-induced ICD by activating ER stress. (a) Curcumin increased the expressions of ER stress-related proteins in normoxic and hypoxic glioma. Cells were irradiated with 10 Gy/25 Gy IR after treatment with or without 30 *μ*M curcumin for 24 h, and then, indicated protein expressions were assessed by WB. (b) Cell apoptosis was assessed with or without pretreatment with 2 *μ*M GSK2606414 or 10 *μ*M 4u8c for 1 h before irradiation. (c) Calreticulin exposure was analyzed by flow cytometry. ^∗^*P* < 0.05, ^∗∗^*P* < 0.01, and ^∗∗∗^*P* < 0.001 vs. control; ^#^*P* < 0.05 and ^##^*P* < 0.01 vs. IR.

ICD is related to ER stress, which is controlled by three unfolded protein response (UPR) sensors, protein kinase RNA-like ER kinase (PERK), inositol-requiring protein 1-*α* (IRE1-*α*), and activating transcription factor 6 (ATF6) [8]. To investigate the role of ER stress in IR-induced ICD, we used GSK2606414, a specific pharmacological inhibitor of PERK phosphorylation, and 4*μ*8c, a specific inhibitor of IRE1-*α*, to block UPR. As shown in Figure 3(a) and Supplementary Figure 1, treatment with curcumin for 24 hours prior to IR significantly increased PERK and IRE1*α* phosphorylation, as well as CHOP and XBP1 protein expression compared to IR alone. Hypoxia induced mild ER stress in glioma cells. These results indicated that PERK and IRE1*α* signaling pathways were activated by IR, which could be enhanced by curcumin. Furthermore, the addition of ER stress inhibitors reduced apoptosis and calreticulin exposure on the cell surface (Figures 3(b) and 3(c)). These results suggested that curcumin enhance IR-induced ICD via ER stress PERK and IRE1*α* signaling pathways.

### 3.4. Curcumin Augmented Chemotaxis and Phagocytosis of DCs to Irradiated Glioma Cells

**Figure 4:**
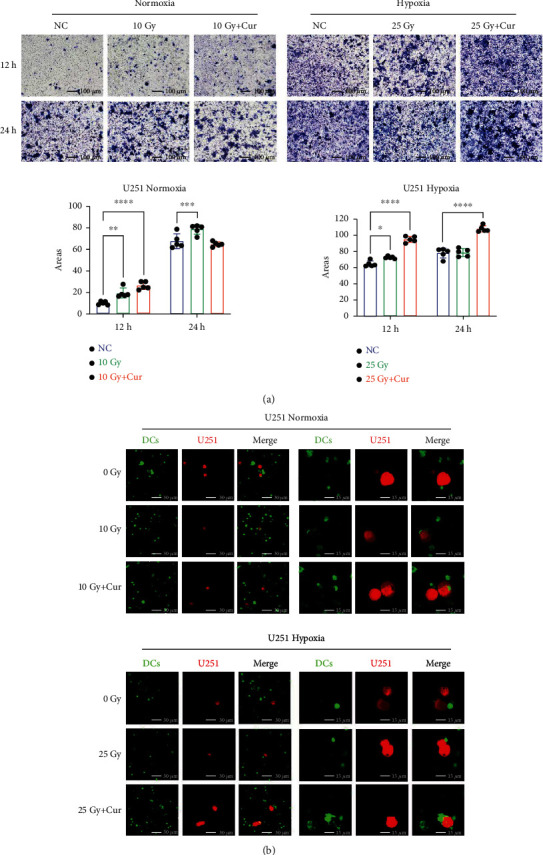
Curcumin augmented chemotaxis and phagocytosis of DCs to irradiated glioma cells. (a) U251 cells treated jointly by curcumin and IR attracted more DCs than the cells treated by IR alone. (b) Curcumin accelerated the recognition and phagocytosis of irradiated glioma cells by DCs. Cells in green represents DC cells, while cells in red represents U251 cells. The magnification of the photos on the right is twice bigger than the left ones.

U251 cells treated jointly by curcumin and IR attracted more DCs than the cells treated by IR alone (Figure 4(a)). The enhancement effect of curcumin on chemotaxis of DCs to irradiated glioma cells was observed at 12 h after irradiation and was more obvious at 24 h after irradiation under hypoxic condition. Next, DCs were labeled green and irradiated U251 cells were labeled red, and then, they were intermixed directly into complete medium to imitate the real intercellular response. As observed in Figure 4(b), DCs began to attached to U251 cells treated with IR alone; meanwhile, cells in the curcumin+IR group could be detected swallowed by DCs after coculture in normoxic or hypoxic condition for 8 h. These results suggested that curcumin accelerate the recognition and phagocytosis of irradiated glioma cells by DCs.

### 3.5. Curcumin Enhanced IR-Induced Glioma ICD In Vivo

**Figure 5:**
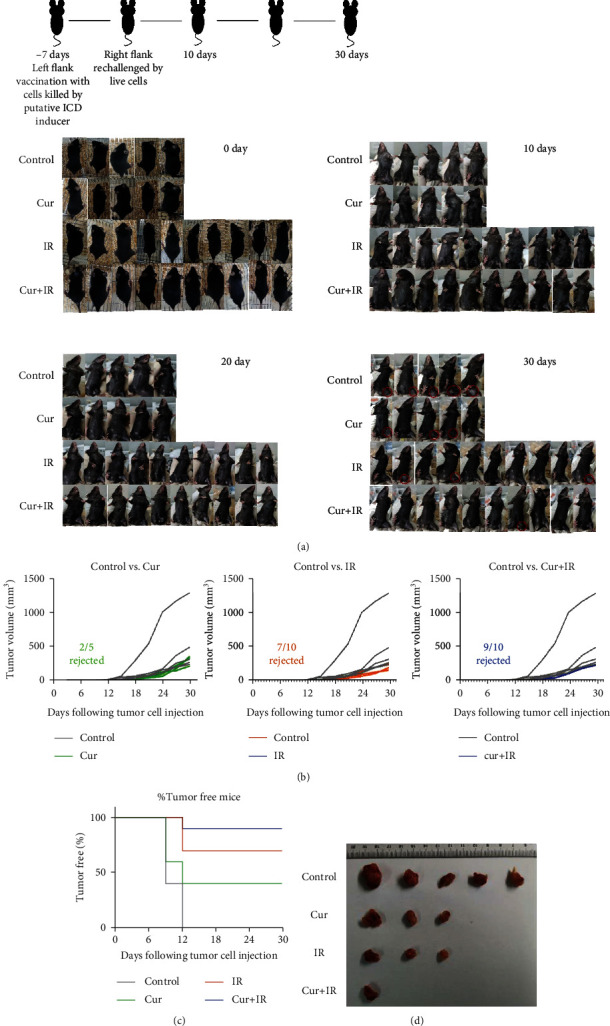
Curcumin enhanced IR-induced glioma ICD in vivo. (a) The schematic diagram and schedules of vaccination experiment. Tumors formed subcutaneously in mice 30 days after inoculation were shown in red circles. (b) Mice were subcutaneously injected with and glioma cells treated with 30 *μ*M curcumin, 10 Gy X-ray, and both, respectively, and the control group was injected with PBS. One week later, tumor cells were injected into the contralateral side, and the tumor size was measured every 3 days. Each line represents a tumor-forming mouse. (c) Kaplan-Meier curves for the percentage of tumor free mice after inoculation with live tumor cells. All treatments significantly delayed or rejected tumor growth compared to control. ^∗^*P* < 0.05, ^∗∗^*P* < 0.01, and ^∗∗∗^*P* < 0.001 vs. control; ^#^*P* < 0.05, and ^##^*P* < 0.01 vs. IR. (d) Tumor photos 30 days after inoculation.

In order to determine whether IR combined with curcumin can induce ICD in glioma, vaccination experiments in mouse models are required, which is the gold standard method for ICD [14]. Glioma cells treated with curcumin and/or X-rays were injected into the left hind limb of immunocompetent syngeneic mice subcutaneously. One week later, glioma cells were injected into the opposite hind limb, and mice were monitored for the rate of tumor formation and tumor size. As shown in Figure 5(b), it was found that all (5/5) mice in the control group developed tumors 12 days after inoculation with live tumor cells, with a tumor formation rate of 100%. Compared with the control group, X-ray-irradiated glioma cells induced antitumor immunity in 70% (7/10) of the mice, rejecting tumor formation. Compared with IR alone, 90% (9/10) of the mice treated with the combination of IR and curcumin did not form tumors, and the tumors that did form appeared later and were smaller than those in the control group (Figures 5(c) and 5(d)). These data indicated that the combination treatment of IR and curcumin induced stronger tumor immune rejection in mice than IR alone, suggesting that curcumin enhance IR-induced glioma ICD in vivo.

### 3.6. Curcumin Enhanced IR-Mediated Immune Cell Infiltration in Tumor Tissue

**Figure 6:**
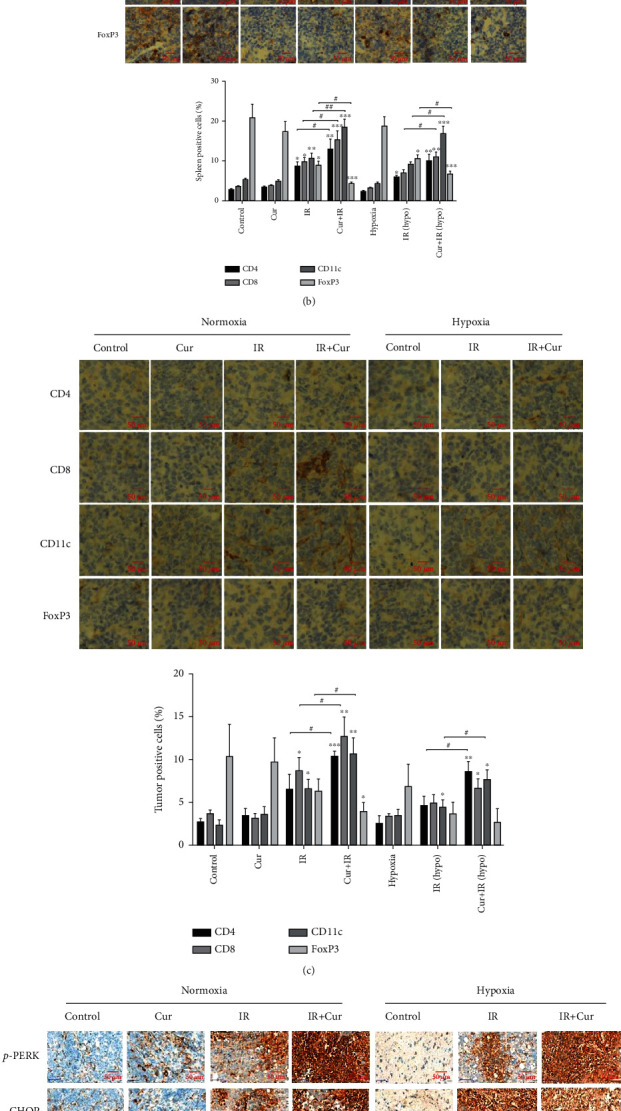
Curcumin enhanced IR-mediated immune cell infiltration in tumor tissue. (a) Mice with normoxic or hypoxic tumor grafts were treated with curcumin and X-ray, and then, tumor and spleen cells were collected. CD3+ and CD8+ T cells were detected by flow cytometry. The number of CD8+ T cells per 10,000 cells was counted. (b) For immunohistochemical staining, immune subsets were shown using anti-CD4, CD8, CD11c, and FoxP3 antibodies in the spleen. (c) For immunohistochemical staining, immune subsets were shown using anti-CD4, CD8, CD11c, and FoxP3 antibodies in tumor tissues. (d) ER stress marker p-PERK, CHOP, p-IRE1*α*, and XBP1 expression were detected by immunohistochemical staining in tumor tissues. ^∗^*P* < 0.05, ^∗∗^*P* < 0.01, and ^∗∗∗^*P* < 0.001 vs. control; ^#^*P* < 0.05, ^##^*P* < 0.01, and ^###^*P* < 0.001 vs. IR.

As shown in Figure 6(a), curcumin treatment before local irradiation of the tumor site induced the production of more CD8+ T cells in the spleen, and the number of tumor-infiltrating CD8+ T lymphocytes was also significantly increased. However, tumor-infiltrating CD8+ T cells did not increase in the hypoxic tumor model, probably due to the killing effect of high-dose radiation on T lymphocytes. Next, we detected the CD4+ and CD8+ T cell subsets, as well as the dendritic cell marker CD11c and regulatory T cell (Treg) marker FoxP3 expression in spleen tissues and tumor tissues by immunohistochemistry. As shown in Figure 6(b), curcumin further increased IR-induced infiltration of CD4+ T cells, CD8+ T cells, and CD11c+ dendritic cells in the spleen tissues and decreased immunosuppressive FoxP3+ regulatory T cells. In normoxic tumor tissues (Figure 6(c)), the infiltration of CD4+ T cells, CD8+ T cells, and CD11c+ dendritic cells was more significant in the combined treatment group than the IR alone group, while the infiltration of FoxP3+ regulatory T cells showed no significant changes. In the hypoxic xenograft tissues, CD4+ T cells and CD11c+ dendritic cells were increased in the combined treatment group than the IR alone group, but CD8+ T cells and FoxP3+ regulatory T cells showed no significant changes between them. As shown in Figure 6(d), curcumin further increased IR-induced ER stress marker p-PERK, CHOP, p-IRE1*α*, and XBP1 expression in both normoxic tumor tissues and hypoxic tumor tissues. This in vivo result verified the role of ER stress signaling in curcumin's enhancing effect on IR-induced ICD in glioma.

## 4. Discussion

Glioma is a common primary intracranial tumor with a 5-year relative survival rate of about 5% [15]. Although surgery, chemotherapy, radiotherapy, and other treatments can be used, glioma is still one of the poorest prognosis malignant tumors due to its high invasiveness and recurrence [16]. Curcumin is a kind of natural flavonoid, which can not only reduce the toxicity of radiotherapy to normal tissues but also increase the radiotherapy-induced death of cancer cells [17, 18]. Curcumin can make glioma and other tumors sensitive to radiotherapy and chemotherapy drugs [19]. This study proved that curcumin could increase the radiation-induced apoptosis in normoxic or hypoxic glioma cells.

ICD is a special regulated apoptotic cell death [20, 21]. Different from the general case in which apoptosis is immune silences, this type of apoptosis induces ROS production and ER stress by some chemical and physical factors and releases certain molecules to activate the immune system [22, 23]. ICD is accompanied by exposure or release of DAMPs including CRT, ATP, and HSP70/90 [22, 24–27]. These molecules can recruit antigen presenting cells (APC) to deliver antigens to the host immune system and initiate antitumor immunity [6, 28]. Our experimental results showed that curcumin significantly enhanced IR-induced calreticulin exposure, which was a powerful “eat me” signal that interacted with phagocytes to assist in phagocytosis of cancer cells [24]. We also found that IR could induce ATP and HSP70 release from glioma cells and curcumin significantly promoted the IR-induced release. Our results showed that hypoxia induced more CD47 production. It was worth noting that curcumin could reduce CD47 expression on the irradiated glioma cell surface, alleviate the “don't eat me” signal, and help to activate a stronger immune response. Although curcumin was not an immunogenic cell death inducer in itself, it could significantly enhance the irradiation-induced DAMP exposure or release.

The occurrence of ICD requires ER stress. ER function can be disturbed by a variety of pathological stimuli, such as hypoxia, irradiation, oxidative stress, nutrient or glucose deficiency, and viral infection [29]. These disturbances can alter or break endoplasmic reticulum homeostasis, resulting in ER stress and the production of misfolded proteins. In the absence of ER stress, the BiP protein, also known as glucose regulatory protein GRP78, binds to three sensors, PERK, IRE1, and ATF6, keeps these proteins in an inactive state, and prevents them from activating [30]. However, in the break of endoplasmic reticulum homeostasis or Ca2+ imbalance, BiP isolates from these sensors and these three transmembrane proteins are activated through dimerization and autophosphorylation, leading to the activation of downstream UPR signaling pathway [31]. When cells fail to respond to ER stress, apoptosis is activated. The C/EBP homologous protein (CHOP) plays a vital role by regulating antiapoptotic and proapoptotic proteins to induce apoptosis [32, 33]. We found that irradiation induced the activation of PERK and IRE1 signaling pathways and upregulated the downstream proapoptotic transcription factor CHOP and the transcription activator XBP1s. These could be further enhanced by curcumin. GSK2606414, a PERK specific inhibitor, is effective in preventing PERK autophosphorylation [34]. 4*μ*8C can bind to the IRE1 active site and selectively inactivate XBP1 splicing and IRE1-mediated mRNA degradation [35]. We found that cell apoptosis and CRT exposure induced by the combination treatment of curcumin and X-ray reduced after PERK or IRE1 inhibition with corresponding inhibitor. The results indicated that the combination treatment of curcumin and IR induced immunogenic cell death of glioma cells through ER stress PERK-eIF2*α* and IRE1*α*-XBP1 signaling pathways.

Vaccination trials can verify the occurrence of ICD in vivo [14]. This experiment required immune-complete homologous host mice. Tumor cells were treated with a potential ICD inducer in vitro and used as a vaccine and then inoculated with tumor cells 1-2 weeks later to monitor tumor formation rate and tumor size. If there is no tumor, it indicates that antitumor immune rejection has occurred in vivo. The ability of mice to reject tumors represents the degree of ICD [7, 36, 37]. Because the traditional in vitro hypoxia method (1% O_2_, 5% CO_2_, and 94% N_2_) was not suitable for hypoxic tumor bearing mouse model, we used cobalt chloride (CoCl_2_) chemical simulation to induce hypoxia in vivo. CoCl_2_ can stabilize HIF-1*α* and produce a reaction similar to hypoxia [38]. Therefore, as a drug to simulate hypoxia, CoCl_2_ is often used to induce hypoxia in vitro and in vivo [39]. We confirmed the occurrence of irradiation-induced ICD in glioma cells through in vivo vaccination experiments, and curcumin could further enhance irradiation-induced ICD via ER stress pathways and produce immune response. Compared to irradiation alone, the combination treatment of curcumin and radiation promoted the infiltration of CD4+ T cells, CD8+ T cells, and CD11c+ dendritic cells in the spleen and tumor tissues of mice and effectively enhanced the antitumor immunity.

## 5. Conclusions

In conclusion, IR combined with curcumin induced normoxic or hypoxic glioma cells ICD in vitro and in vivo, providing a new avenue for enhancing DC activation and antitumor immunity. These data provide a rationale for curcumin combined with radiotherapy and/or immunotherapy for glioma patients who failed conventional radiotherapy and chemotherapy.

## Data Availability

Research data are stored in an institutional repository and will be shared upon request to the corresponding author.

## Supplementary Materials

Supplementary Materials Supplementary Figure 1: full membrane western blot images of ER stress PERK and IRE1*α* signaling pathway-associated proteins.
