# Supplementary material for: Curcumin Enhanced Ionizing Radiation-Induced Immunogenic Cell Death in Glioma Cells through Endoplasmic Reticulum Stress Signaling Pathways
Source: Oxid Med Cell Longev. 2022 Oct 4;2022:5424411. doi: 10.1155/2022/5424411 (PMC9553401; doi:10.1155/2022/5424411)

Supplementary Figure 1

Full membrane Western Blot images of the proteins in Figure 3A

p-PERK
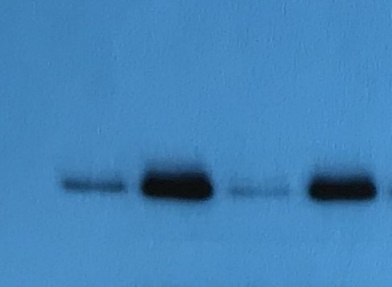

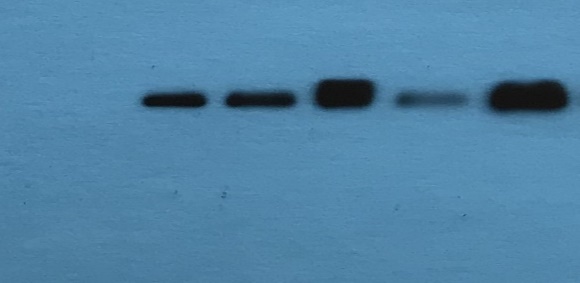


PERK
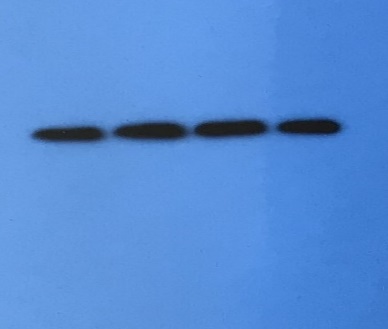

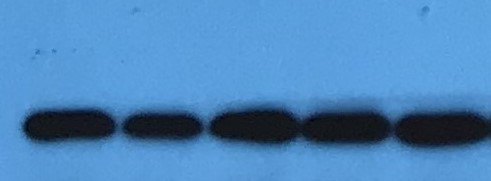


CHOP
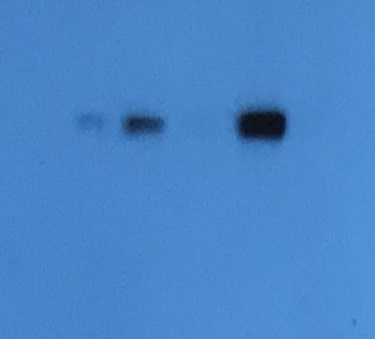

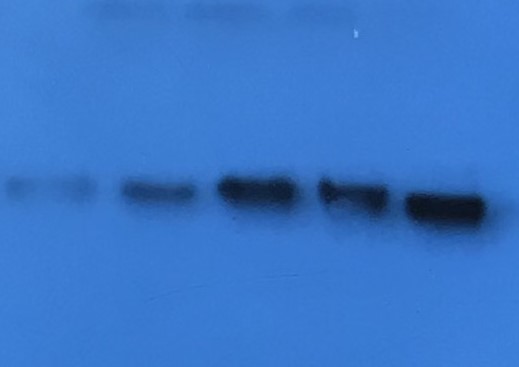


p-IRE1-α
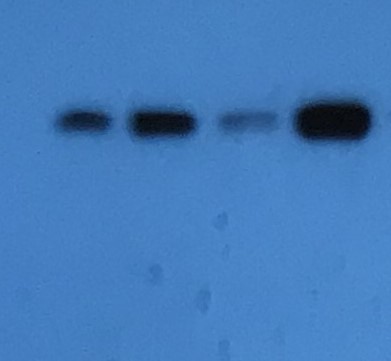

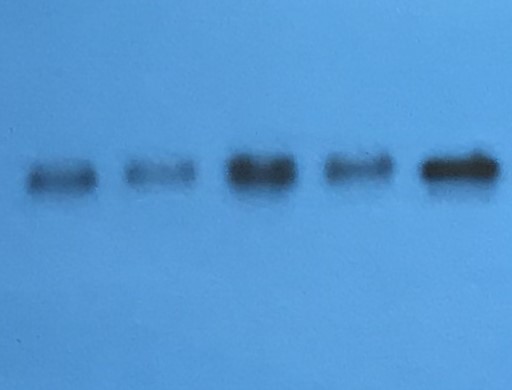


IRE1-α
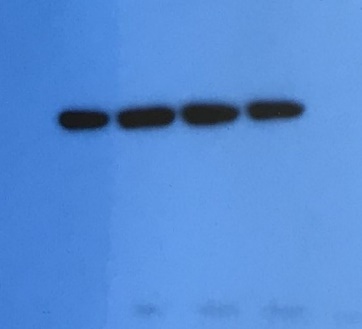

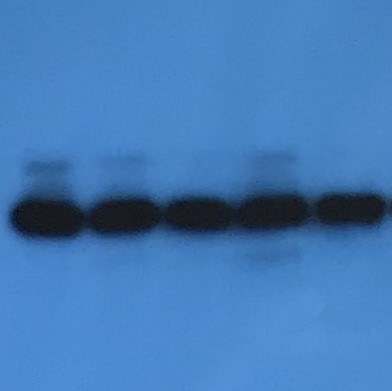


XBP1s
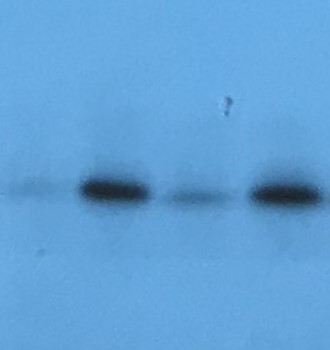

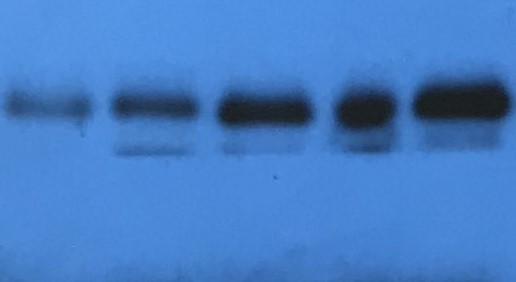


Actin
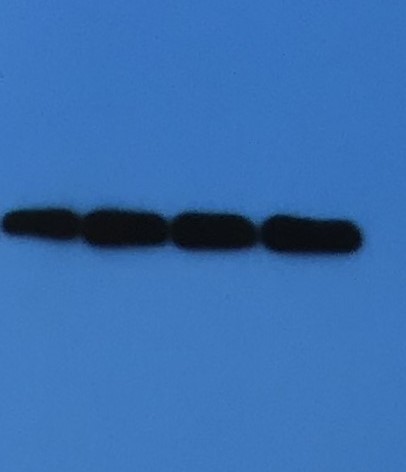

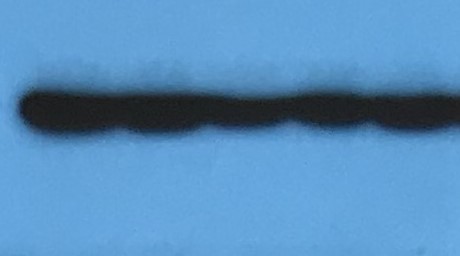

Supplement: Supplementary Materials — Supplementary Figure 1: full membrane western blot images of ER stress PERK and IRE1α signaling pathway-associated proteins. [file 5424411.f1.docx]
